# Structural networks involved in attention and executive functions in multiple sclerosis

**DOI:** 10.1016/j.nicl.2016.11.026

**Published:** 2016-12-05

**Authors:** Sara Llufriu, Eloy Martinez-Heras, Elisabeth Solana, Nuria Sola-Valls, Maria Sepulveda, Yolanda Blanco, Elena H. Martinez-Lapiscina, Magi Andorra, Pablo Villoslada, Alberto Prats-Galino, Albert Saiz

**Affiliations:** a Center of Neuroimmunology, Laboratory of Advanced Imaging in Neuroimmunological Diseases, Hospital Clinic Barcelona, Institut d'Investigacions Biomediques August Pi i Sunyer (IDIBAPS) and Universitat de Barcelona, Barcelona, Spain; b Laboratory of Surgical NeuroAnatomy (LSNA), Facultat de Medicina, Universitat de Barcelona, Barcelona, Spain

**Keywords:** MRI, Connectivity, Tractography, Graph analysis, Multiple sclerosis, Cognition

## Abstract

Attention and executive deficits are disabling symptoms in multiple sclerosis (MS) that have been related to disconnection mechanisms. We aimed to investigate changes in structural connectivity in MS and their association with attention and executive performance applying an improved framework that combines high order probabilistic tractography and anatomical exclusion criteria postprocessing. We compared graph theory metrics of structural networks and fractional anisotropy (FA) of white matter (WM) connections or edges between 72 MS subjects and 38 healthy volunteers (HV) and assessed their correlation with cognition. Patients displayed decreased network transitivity, global efficiency and increased path length compared with HV (*p* < 0.05, corrected). Also, nodal strength was decreased in 26 of 84 gray matter regions. The distribution of nodes with stronger connections or hubs of the network was similar among groups except for the right pallidum and left insula, which became hubs in patients. MS subjects presented reduced edge FA widespread in the network, while FA was increased in 24 connections (*p* < 0.05, corrected). Decreased integrity of frontoparietal networks, deep gray nuclei and insula correlated with worse attention and executive performance (r between 0.38 and 0.55, *p* < 0.05, corrected). Contrarily, higher strength in the right transverse temporal cortex and increased FA of several connections (mainly from cingulate, frontal and occipital cortices) were associated with worse functioning (r between − 0.40 and − 0.47, *p* < 0.05 corrected). In conclusion, structural brain connectivity is disturbed in MS due to widespread impairment of WM connections and gray matter structures. The increased edge connectivity suggests the presence of reorganization mechanisms at the structural level. Importantly, attention and executive performance relates to frontoparietal networks, deep gray nuclei and insula. These results support the relevance of network integrity to maintain optimal cognitive skills.

## Highlights

• High order tractography and anatomical exclusion criteria improve connectivity analyses.
• Structural connectivity is less efficient in multiple sclerosis.
• Attentional and executive functions relate to integrity of strategic networks.
• Increased connectivity suggests structural reorganization mechanisms.

## 1 Introduction

Cognitive dysfunction is present in 40%–70% of patients with multiple sclerosis (MS) (Amato et al., 2010) predominantly affecting attention and executive functions, information processing speed and episodic memory (Rocca et al., 2015). Attention and executive functions are critical cognitive domains to successfully carry out daily life activities and social functioning. The Paced Auditory Serial Addition Test (PASAT) and the Symbol Digit Modalities Test (SDMT) are sensitive tools to examine different components of attention, executive functions and information processing speed and are widely used in neuropsychological evaluation of patients with MS (Boringa et al., 2001, Cutter et al., 1999). Cognitive dysfunction in MS has been associated with disconnection mechanisms related to focal lesions and diffuse microstructural damage in strategic white matter (WM) tracts involved in cognitive performance, in addition to gray matter (GM) impairment (Bozzali et al., 2013, Calabrese et al., 2009, Harrison et al., 2015, Llufriu et al., 2014, Preziosa et al., 2016). Moreover, brain functional modifications in task-based studies seem to drive compensatory mechanisms at the earliest stages of disease, when structural damage is low, with increased cortical recruitment probably contributing to the maintenance of a normal level of cognitive performance. In later phases, those changes are lost or exhausted leading to cognitive dysfunction (Rocca et al., 2015).

Recently, graph theoretical approaches have been applied to the characterization of complex networks in the brain (He et al., 2009, Rocca et al., 2016, Sporns et al., 2005). Functional reports have shown varying patterns of connectivity, with increased or decreased connectivity in MS subjects with cognitive impairment (Schoonheim et al., 2015). All in all, the controversy of those changes as being beneficial or maladaptive is still unsolved. In addition, structural connectivity through diffusion tensor imaging (DTI) tractography showed a reduction of network efficiency from early stages of disease that correlated with physical disability (Li et al., 2013, Shu et al., 2011). DTI-based fiber tractography have promoted the development of structural connectivity techniques, which define and quantify anatomical links between remote GM regions by WM fiber pathways (Guye et al., 2010). However, it presents a set of challenges to be overcome (Jones, 2010) as most methods result in systematically unreliable and anatomically misleading information that can be particularly serious in the presence of lesions such as in MS (Farquharson et al., 2013). High order probabilistic tractography based on second order integration over fiber orientation distributions (iFOD2) estimated with constrained spherical deconvolution (CSD) is able to improve accuracy in fiber tracking (Tournier and Connelly, 2010) but has not been widely adopted in structural connectivity analyses. Albeit, tractography generates biologically unrealistic streamlines that can be reduced by the application of an anatomical exclusion criteria (AEC) postprocessing. AEC removes streamlines of low confidence and excludes pathways reaching GM areas outside the seeds masks, reducing the amount of anatomically implausible streamlines and increasing tractography reliability in healthy volunteers (HV) and in patients with MS (Martínez-Heras et al., 2015).

The relevance of several features of the MS pathology, such as demyelination and axonal injury and repair, and their influence on cognition are still unknown. In this way, the characterization of the brain structural network has the potential to improve our understanding of the pathophysiology of MS and its clinical manifestations. In this study, we aimed to investigate changes in structural connectivity in patients with MS and their association with attention and executive functioning applying an improved framework for tractography reconstruction by combining iFOD2 and automatic postprocessing based on AEC (Martínez-Heras et al., 2015). We hypothesized that the disruption of the structural network connectivity contributes to cognitive difficulties characteristics of this disease, especially in attention and executive functions. In order to study our hypothesis, we evaluated the changes in whole brain graph theory metrics, WM connections (edges) and GM regions (nodes) and their correlation with cognition in a set of MS subjects. Moreover, we assessed the reproducibility (Bonilha et al., 2015) of the structural networks from 15 HV in order to evaluate the reliability of our framework.

## 2 Material and methods

### 2.1 Participants

MS participants were consecutively recruited from the MS Unit at the Hospital Clinic of Barcelona and they had to be free from relapses in the last 30 days. Other inclusion criteria were as follows: (a) aged between 18 and 65 years; (b) an Expanded Disability Status Scale (EDSS) (Kurtzke, 1983) of 6.0 or lower; and (c) have no significant medical illness that could interfere with cognitive functioning. We included 72 subjects with relapsing-remitting or secondary-progressive MS according to 2010 McDonald criteria (Polman et al., 2011) and 38 HV without previous or present history of neurological or psychiatric dysfunction. HV were recruited from the outpatient facility of the Neurology department via advertisements. The Ethics Committee of the Hospital Clinic of Barcelona approved the study and all participants signed an informed consent.

### 2.2 Cognitive evaluation

Cognitive function was assessed with PASAT and SDMT in MS participants. Three second-PASAT is a mental simple calculation test involving attention, auditory working memory, and information processing speed (Gronwall, 1977), while SDMT is a symbol-number substitution test designed to assess information processing speed and visual attention (Smith, 1968). Cognitive evaluations were performed by a neuropsychologist (ES) blinded to MRI and other clinical data. Test z-scores were derived from normative data obtained from a published healthy cohort and were stratified by age and education (Sepulcre et al., 2006). z-Scores were calculated for each test (zPASAT and zSDMT) and a mean attentional-executive domain z-score (zAttention) was obtained (Sepulcre et al., 2006).

### 2.3 MRI data acquisition

MRI images were acquired on a 3 T Magnetom Trio (SIEMENS, Erlangen, Germany) scanner, using a thirty-two channel phased-array head coil and included 3D Magnetization Prepared Rapid Acquisition Gradient Echo (MPRAGE) structural and diffusion weighted imaging (DWI) sequences. The 3D-structural image had the following acquisitions parameters: TR, 1800 ms; TE, 3.01 ms; TI, 900 ms; 240 contiguous sagittal slices with 0.94 mm isotropic voxel size; 256 × 256 matrix size. The DWI was a High Angular Resolution Diffusion Imaging (HARDI) sequence with TR/TE, 14,800/103 ms; 100 contiguous axial slices; 1.5 mm isotropic voxel size; 154 × 154 matrix size; b value, 1000 s/mm^2^; 60 diffusion encoding directions and a single baseline image acquired at 0 s/mm^2^.

Parallel imaging was applied with a geometric reduction factor of 2 to reduce the distortion caused by susceptibility differences at tissue interfaces. In addition, a fieldmap sequence (TE1, 4.92 ms and TE2, 7.38 ms, with the same slice prescription, slice thickness and field of view as the HARDI sequence) was acquired to correct geometric distortions of the DWI.

In order to assess the reproducibility of the tractography framework, 5 HV underwent a second MRI acquisition on the same day (intra-session reproducibility) and 10 HV on different days (inter-session reproducibility).

### 2.4 Reconstruction of structural networks

#### 2.4.1 Anatomical parcellation

Cortex was parcellated in 3D-structural image with Freesurfer (FS) software V5.3.0 (http://freesurfer.net/) in 34 regions per hemisphere with the Desikan-Killiany atlas (Desikan et al., 2006). Subcortical GM regions (8 per hemisphere) were segmented with FIRST tool (Patenaude et al., 2011) as it is more reliable for these structures than FS segmentation (Morey et al., 2010). Whole GM volume was calculated in each subject. Additionally, a manual mask of WM lesions delineated with the ITK-SNAP V3.4 toolkit (http://www.itksnap.org/pmwiki/pmwiki.php) on the 3D-structural image was used for lesion inpainting to improve segmentation and registration steps in MS participants (Sdika and Pelletier, 2009).

#### 2.4.2 Whole brain tractography

**Fig. S1:**
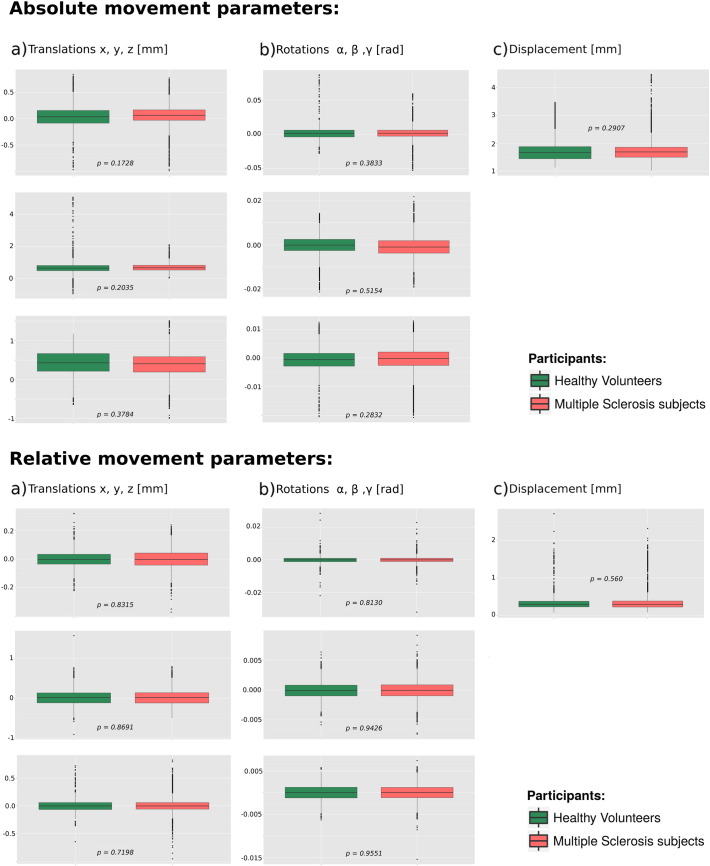
Box plots of absolute motion values obtained from b0 reference volumes and relative motion values from preceding volumes of diffusion weighted images.

**Fig. 1:**
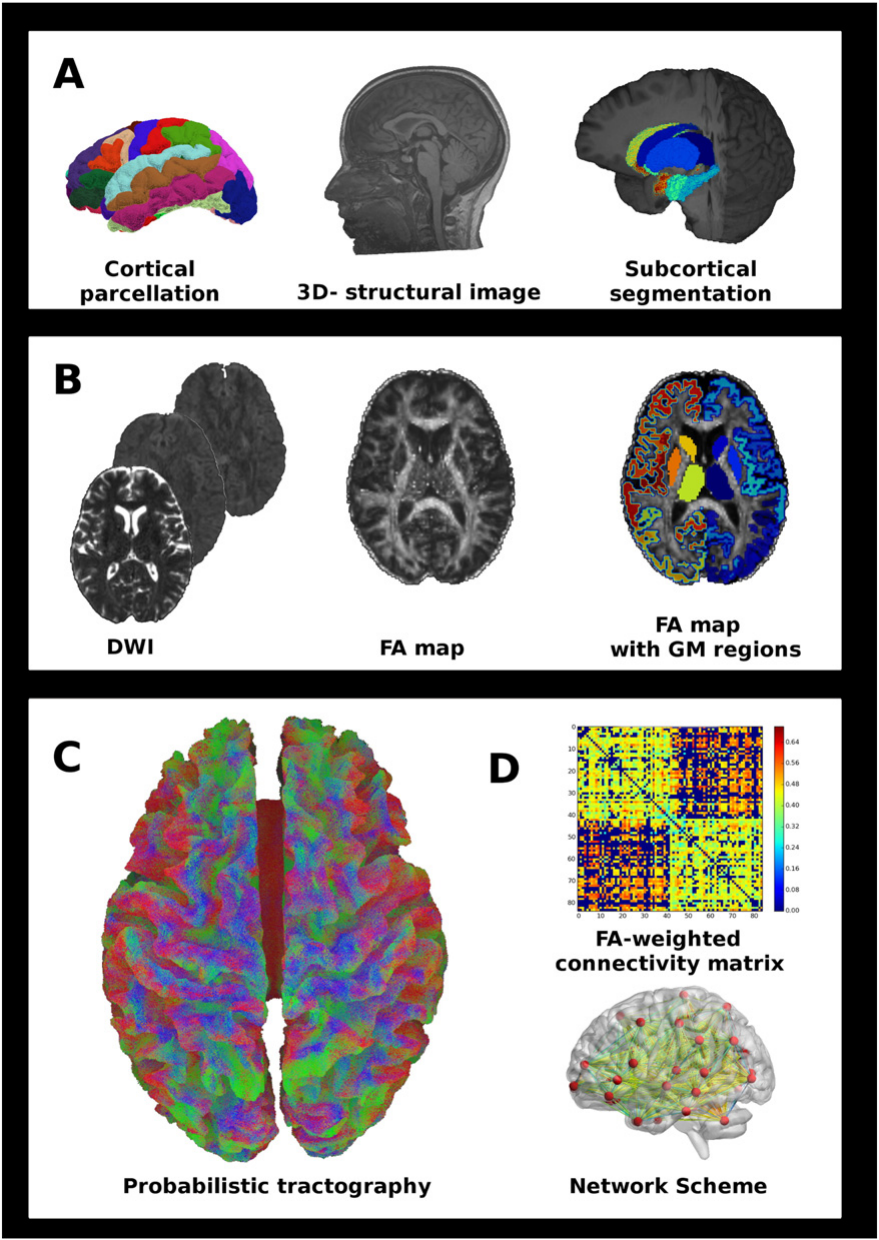
Structural connectivity framework. A) Nodes parcellation: Freesurfer and FIRST tools were used to segment the cortex and the subcortical GM areas from the 3D-structural image. B) DWI processing: FA maps were obtained through standard preprocessing of DWI. Then, subcortical and cortical parcellations were registered to FA maps. C) Diffusion tractography: Probabilistic streamline fiber tracking by iFOD2 including anatomical exclusion criteria postprocessing. D) Structural network reconstruction: Connections were assembled into FA-weighted connectivity matrices. Network scheme included 84 cortical and subcortical GM nodes and FA edges linking pairs of nodes. Several graph theory metrics were computed from the resulting matrices. FA: fractional anisotropy; DWI: diffusion weighted images; iFOD2: second order integration over fiber orientation distributions; GM: gray matter.

The schematic diagram of the framework is shown in Fig. 1. Standard preprocessing of the DWIs included geometric distortion correction for echo planar images (EPI) with fieldmap images and head motion correction. Fieldmap-based unwarping of the EPI was done with PRELUDE to unwrap the phase and FUGUE to compute the distortion by means of FMRIB Software Library (FSL V5.0, http://fsl.fmrib.ox.ac.uk/fsl/fslwiki/). We evaluated data quality from each participant by visual inspection of the DWIs. Besides, we calculated head motion through DTI Motion tool (https://github.com/Woutervdbos/DTI-Motion) and did not find any significant difference in absolute and relative movement parameters between HV and MS participants (Supplementary material, Fig. S1). After the application of eddy current correction, the gradient vectors of the DWIs were rotated to compensate head motion. The 84 segmented regions in GM were then registered to undistorted EPI (Greve and Fischl, 2009) and used as nodes in the network setup.

The estimation of the diffusion signal response function for each voxel was done to capture the underlying fiber orientations distribution (FOD). The MRtrix3 package (http://www.mrtrix.org/) was used to perform probabilistic fiber tracking by iFOD2. A set of 3 million streamlines in the brain were generated by seeding the whole brain mask. The default step size (0.75), curvature (45°) and FOD amplitude threshold (0.1) were used. Then, the 5-tissue type segmented images (5TT) were provided to the anatomically-constrained tractography (ACT) tool, which improves tractography reconstruction using anatomical information through a dynamic thresholding strategy (Smith et al., 2012). In MS participants, normal appearing WM and lesions mask were considered as WM tissue type in the 5TT format. AEC was applied to every track file from each pair of nodes (3486 specific connections of interest linking 84 GM regions) to reduce the presence of false positive streamlines (Martínez-Heras et al., 2015). This included the application of a 1% threshold based on track-weights for each connection and the exclusion of any streamline outside the direct link between each pair of nodes.

A neuroanatomist (AP) visually inspected 3 specific fiber tracks connecting pairs of nodes (fusiform and lateral occipital cortices, putamen and superior frontal cortex, thalamus and precentral cortex) to ensure the reconstruction of these tracks and to detect anatomically implausible streamlines. The chosen connections participate in important functional circuits, are well defined anatomically and their reconstruction is challenging (Nieuwenhuys et al., 2008, Schmahmann and Pandya, 2009).

### 2.5 Network analysis

Brain structural networks were represented by 84 × 84 weighted connectivity matrices. Connectome matrices represented mean fractional anisotropy (FA) values along each connection based on tracking results. Graph analysis was used to express local and global connectivity properties of networks (Liu et al., 2012). Whole brain graph theory measures included strength (the sum of all neighboring link weights), transitivity (the ratio of triangles to triplets in the network), global efficiency (the average inverse shortest path length), assortativity (a correlation coefficient between the degrees of all nodes on two opposite ends of a link), clustering coefficient (the fraction of the node's neighbors that are also neighbors of each other) and betweenness centrality (BC, the fraction of all shortest paths in the network that pass through a given node) (Rubinov and Sporns, 2010, Sporns et al., 2005). Moreover, the strength and BC of each node was calculated. Nodes showing higher strength (above 1 standard deviation, SD, of the mean strength of all nodes in each group of participants) were considered hubs of the network (Van den Heuvel and Sporns, 2011). All measures belonging to graph theory were calculated using the Brain Connectivity Toolbox (https://sites.google.com/site/bctnet/).

### 2.6 Statistics

Intra-session and inter-session reproducibility of FA-weighted connectivity matrices was calculated by intra-class correlation coefficients (ICCs) comparing matrices before and after the application of AEC as it is widely used to contrast tractography approaches (Bonilha et al., 2015). Absolute agreement between FA weighted connectivity matrices from two time points was computed through R statistical software (ICC package) (Wolak et al., 2012). The disparity produced by structural segmentation in different time points was avoided by using the same anatomical segmentation.

Student's *t*-test or Chi-square test was used to compare demographic and clinical characteristics between subjects with MS and HV. We also compared whole brain graph theory metrics and FA-weighted connectivity matrices between both groups through Student's *t*-tests with Bonferroni correction for multiple comparisons (*p* < 0.008). Correlations between nodal strength and edge mean FA with cognitive z-scores was computed using the Pearson correlation coefficient. Statistical significance was set at *p* < 0.05. Due to the large number of nodes and edges included, we applied false discovery rate (FDR) correction to all analyses. Statistics were performed with Matlab (V R2013a) and SPSS (V20.0).

## 3 Results

MS volunteers had a mean (SD) attentional z-score of − 0.14 (1.10). Clinical, demographic and cognitive data from subjects are summarized in Table 1. MS subjects and HV were similar in age and gender.

### 3.1 Assessment of the tractography results

**Fig. S2:**
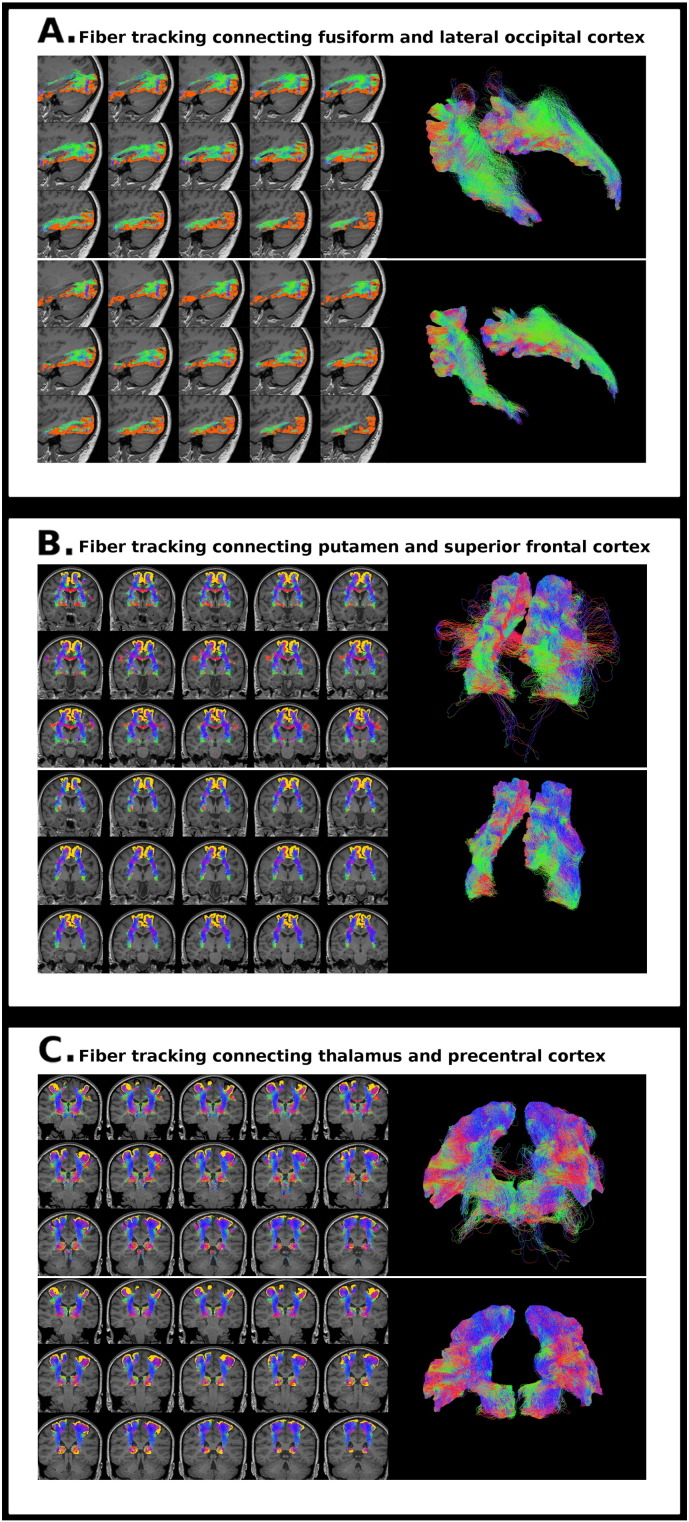
Streamlines of 3 specific fiber tracks connecting pairs of nodes in a healthy volunteer. A) Fusiform and lateral occipital cortices, B) putamen and superior frontal cortex, C) thalamus and precentral cortex. The resulting tractography reconstructions before (upper rows) and after (bottom rows) anatomical exclusion criteria postprocessing are displayed.

**Fig. S3:**
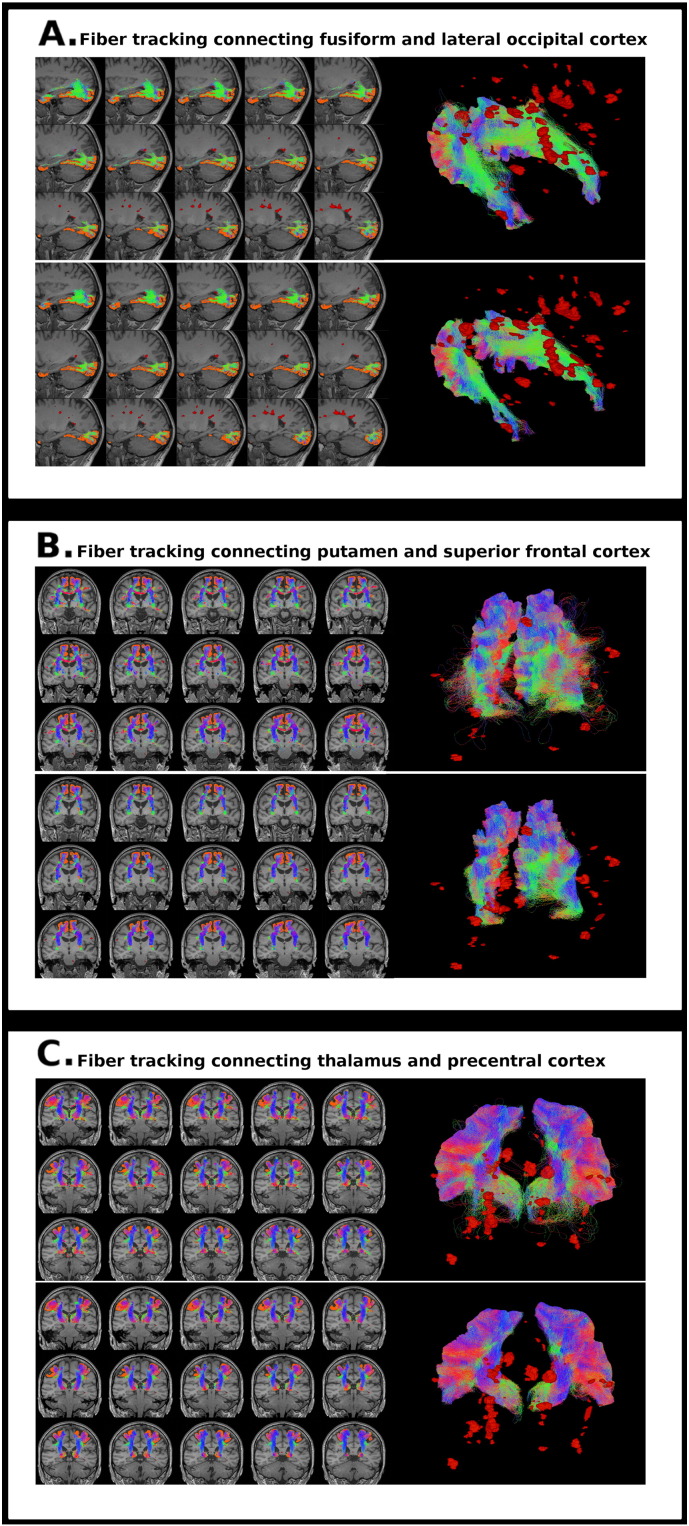
Streamlines of 3 specific fiber tracks connecting pairs of nodes in a subject with MS. A) Fusiform and lateral occipital cortices, B) putamen and superior frontal cortex, C) thalamus and precentral cortex. The resulting tractography reconstructions before (upper rows) and after (bottom rows) anatomical exclusion criteria postprocessing are displayed. Multiple sclerosis lesions are colored in red.

The revised tracks were reconstructed in all subjects. Both in HV and in MS participants, the use of AEC postprocessing removed streamlines containing low values of weights and decreased anatomically unrealistic streamlines in the inspected tracks (see Supplementary material, Figs. S2 and S3).

Intra-session ICCs comparing FA-weighted connectivity matrices before and after AEC postprocessing were evaluated in 5 HVs. Mean (SD) ICCs were 0.77 (0.02) and 0.78 (0.03) respectively, corresponding to an increase of 1.28% in reproducibility.

Mean (SD) inter-session ICCs in 10 HVs were 0.69 (0.07) and 0.74 (0.06) before and after application of AEC, corresponding to an increment of 6.16%. The mean (SD) number of days between the two MRI acquisitions was 53.8 (21.9).

### 3.2 Comparison of structural connectivity metrics between MS subjects and healthy volunteers

Patients showed decreased (*p* < 0.008, Bonferroni correction) whole brain transitivity and global efficiency, and increased path length compared to HV (Table 2). These results remained unchanged when we considered only patients with RRMS.

**Fig. S4:**
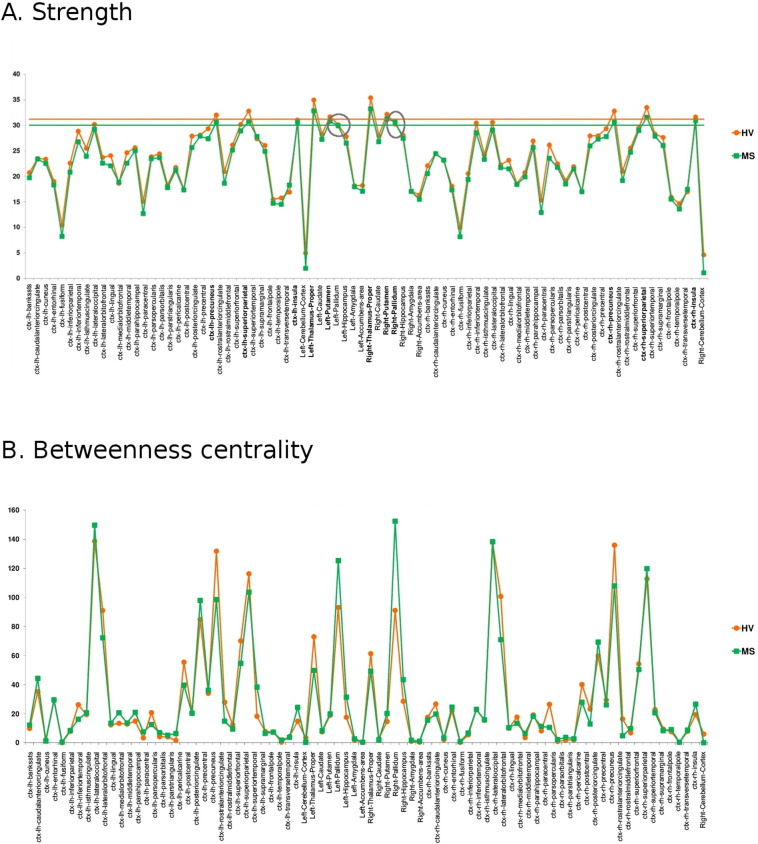
Strength (A) and betweenness centrality (B) of all nodes were calculated in MS subjects and healthy volunteers (HV). Horizontal lines indicate hub threshold (green line for HV group and orange for MS subjects group). Nodes with strength above the threshold were considered hubs and appear highlighted in bold. Hubs indentified only in MS subjects appear inside circles. ctx: cortex; lh: left hemisphere; rh: right hemisphere.

**Fig. 2:**
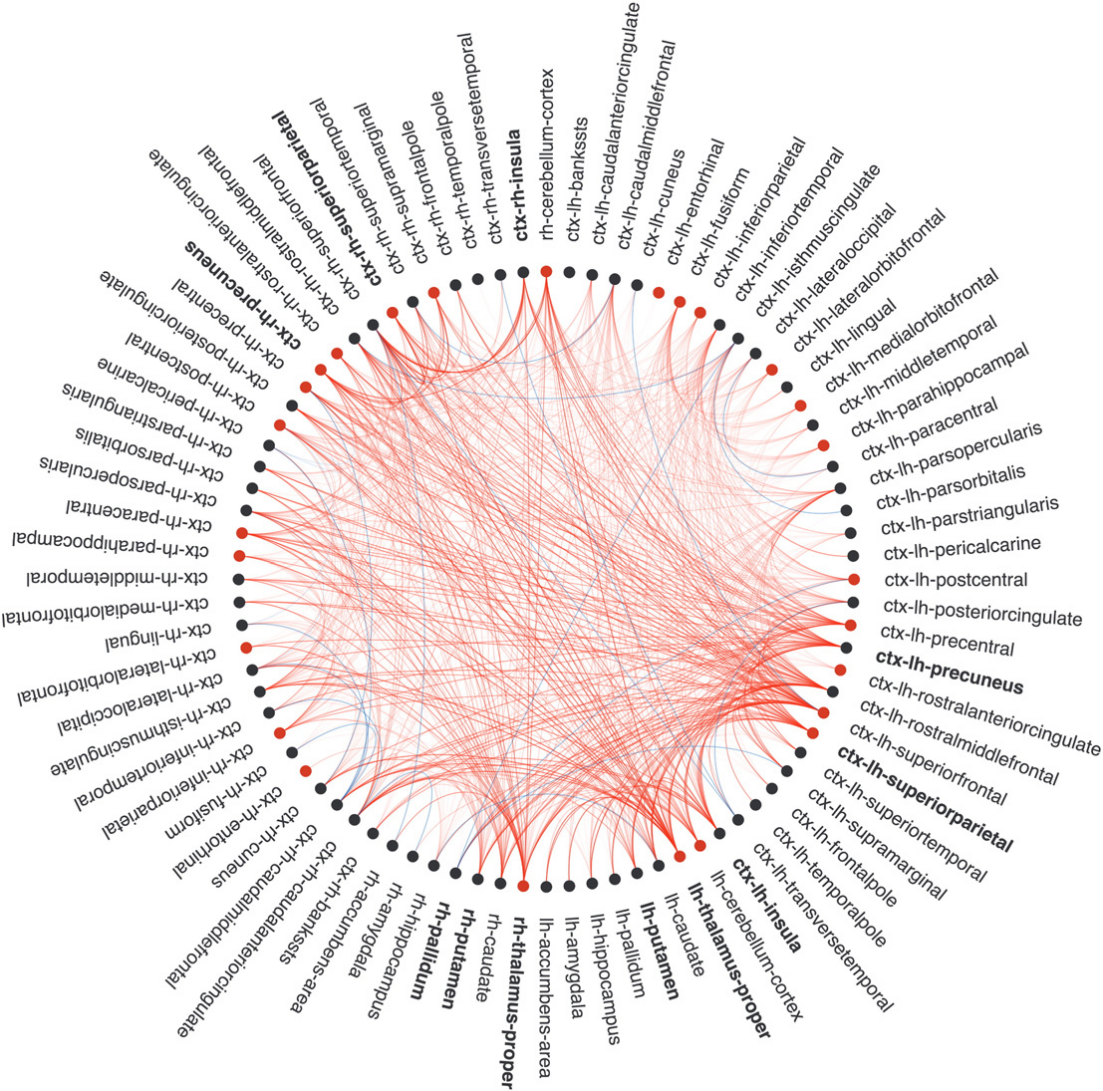
Group comparison for edge FA and nodal strength. Nodes are located in the corresponding vertices of the circle. Only edges showing significant differences (*p* < 0.05, FDR) between MS participants and healthy volunteers are displayed as lines (reduction of edge FA in patients is colored in red and increase in blue). Also, nodes with significant decrease of strength in MS are colored in red. Hub regions in MS are highlighted in bold. ctx: cortex; lh: left hemisphere; rh: right hemisphere. This connectogram was developed by Bokeh from Python v2.7 (http://bokeh.pydata.org/en/latest/).

Nine hubs were identified in both groups and included GM midline regions (bilateral precuneus), bilateral superior parietal cortex, right insula and subcortical nuclei including thalamus and putamen. However, in MS subjects, right pallidum and left insula were also classified as hubs (Fig. 2), and increased their relative BC in the brain network of this group (see Supplementary material, Fig. S4).

In patients, 26 of the 84 nodes showed decreased nodal strength (*p* < 0.05, FDR) compared with HV, corresponding to parietal cortex (bilateral inferior parietal, bilateral postcentral, bilateral superior parietal, right supramarginal gyrus and right precuneus), temporal lobe (left fusiform cortex), frontal and prefrontal cortex (left superior frontal, bilateral precentral, right paracentral, bilateral lateral orbitofrontal and left medial orbitofrontal cortex), bilateral thalamus, limbic lobe (bilateral entorhinal, bilateral parahippocampal and bilateral rostral anterior cingulate) and bilateral cerebellar cortex (Fig. 2).

Patients also presented reduction of FA in 793 edges out of 3486 connections, while FA was increased in 24 edges (i.e. right middle frontal cortex with occipital nodes) (*p* < 0.05, FDR) (Fig. 2). Indeed, FA of connections between the right pallidum (new hub in patients) and left isthmus of the cingulate cortex, postcentral and supramarginal cortices was increased in patients (*p* < 0.05, FDR).

### 3.3 Correlations between attentional-executive functions and structural connectivity measures

**Fig. S5:**
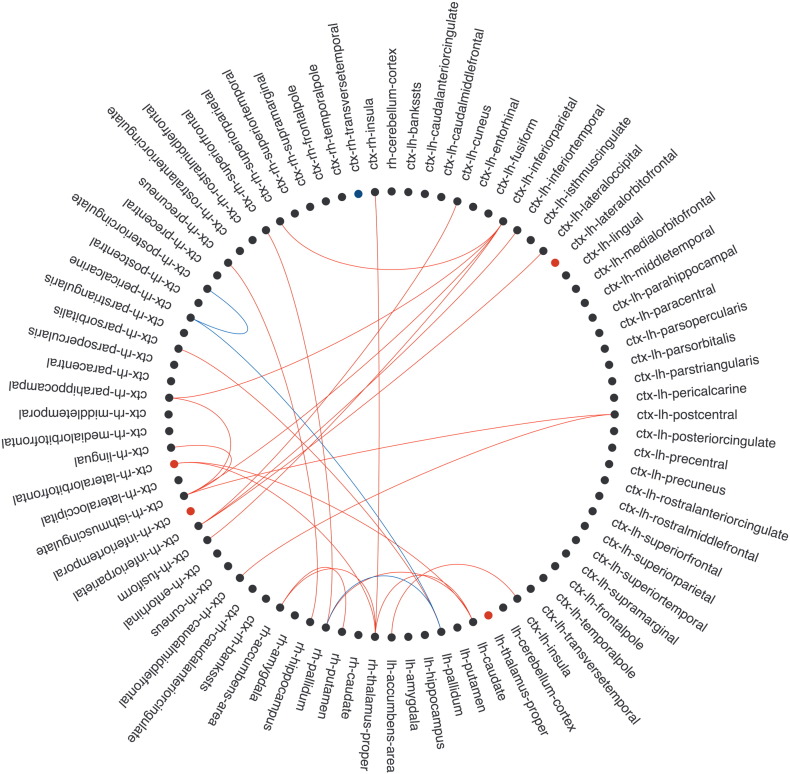
Correlations between edge FA, nodal strength and zAttention in the group of RRMS subjects. Nodes are located in the corresponding vertices of the circle. Edges with significant correlations (*p* < 0.05, FDR) between FA and attentional performance are shown as lines (in red for positive and blue for negative correlations). Also, nodes with significant correlations with z-scores are colored (in red for positive and blue for negative). ctx: cortex; lh: left hemisphere; rh: right hemisphere. This connectogram was developed by Bokeh from Python v2.7 (http://bokeh.pydata.org/en/latest/).

**Fig. 3:**
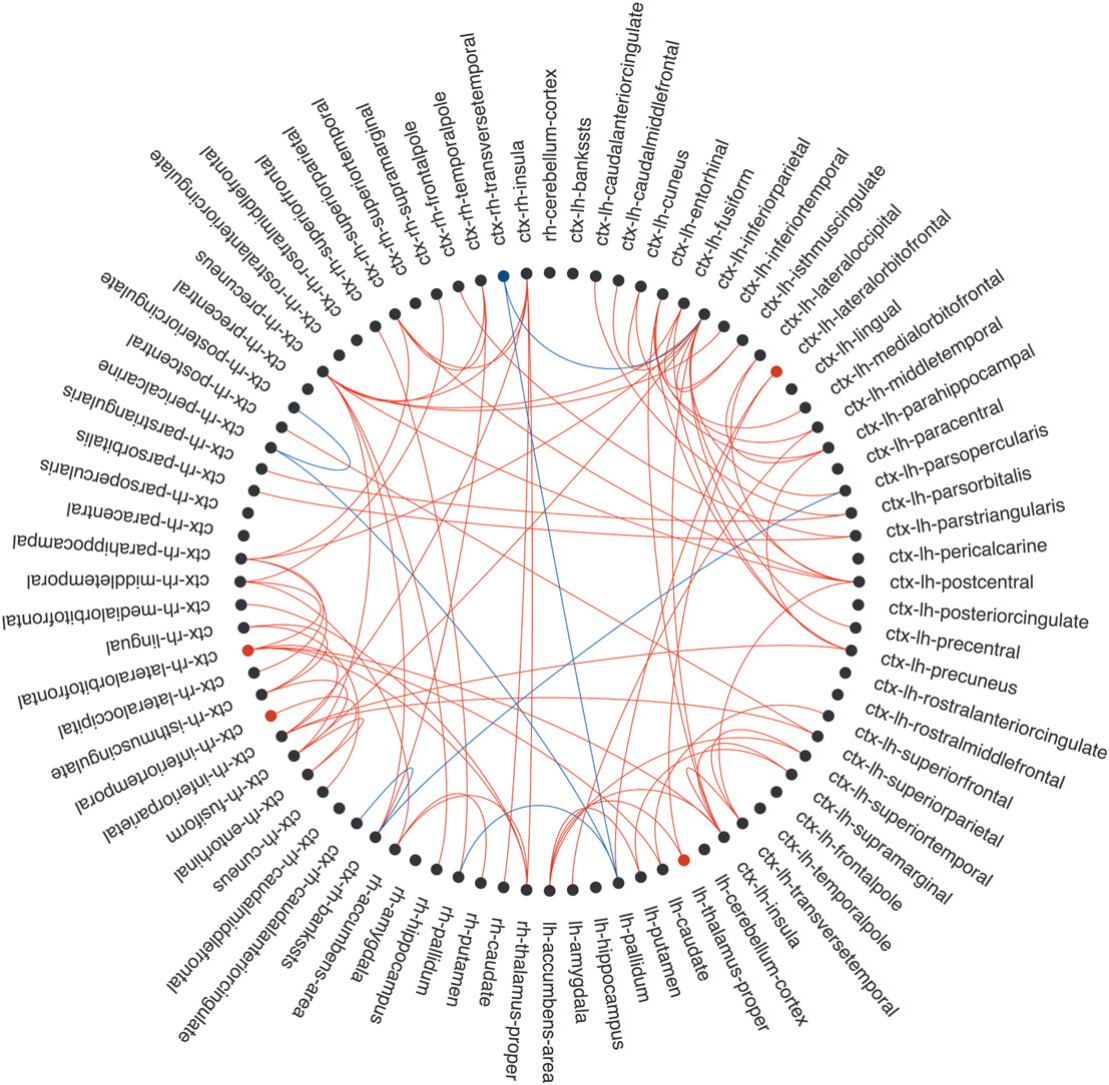
Correlations between edge FA, nodal strength and zAttention. Nodes are located in the corresponding vertices of the circle. Edges with significant correlations (*p* < 0.05, FDR) between FA and attentional performance are shown as lines (in red for positive and blue for negative). Also, nodes with significant correlations with z-scores are colored (in red for positive and blue for negative correlations). ctx: cortex; lh: left hemisphere; rh: right hemisphere. This connectogram was developed by Bokeh from Python v2.7 (http://bokeh.pydata.org/en/latest/).

**Fig. 4:**
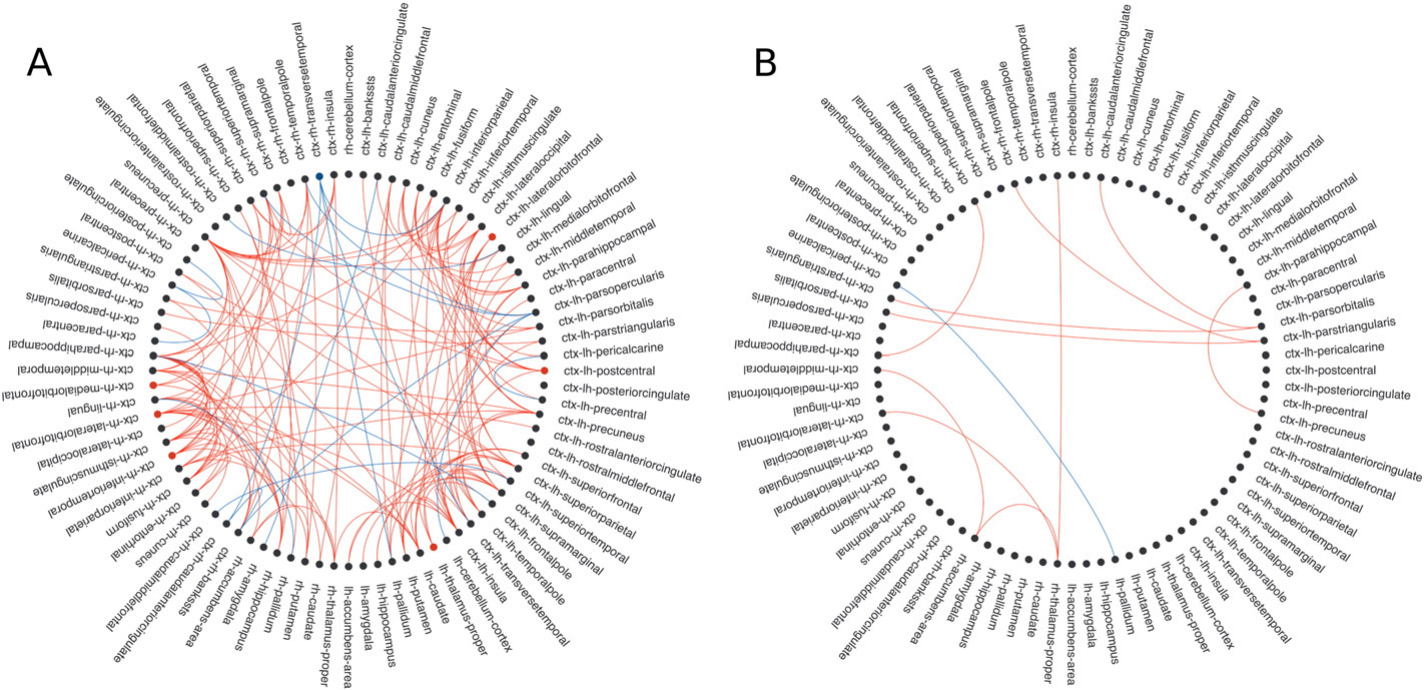
Correlations between edge FA, nodal strength and A) zPASAT or B) zSDMT scores. Nodes are located in the corresponding vertices of the circle. Edges with significant correlations (*p* < 0.05, FDR) between FA and test performance are shown as lines (in red for positive and blue for negative correlations). Also, nodes with significant correlations with z-scores are colored (in red for positive and blue for negative). ctx: cortex; lh: left hemisphere; rh: right hemisphere. These connectograms were developed by Bokeh from Python v2.7 (http://bokeh.pydata.org/en/latest/).

Decreased strength in 4 nodes correlated (r between 0.36 and 0.42, *p* < 0.05, FDR) with worse zAttention including bilateral lateral orbitofrontal, right inferior temporal cortices, and left thalamus (Fig. 3). In addition, increased strength in the right transverse temporal cortex correlated with worse zAttention (*r* = − 0.35, *p* = 0.04, FDR). These correlations remained unchanged in the RRMS group (see Supplementary material, Fig. S5). Nodal strength in 7 regions was associated with zPASAT (Fig. 4A) while correlations between nodal properties and zSDMT did not survive FDR correction (Fig. 4B).

We detected significant positive correlations (r between 0.38 and 0.55, *p* < 0.05, FDR) between decreased FA and worse zAttention in 73 connections. On the contrary, increased FA in 6 connections correlated with worse zAttention (r between − 0.40 and − 0.47, *p* < 0.05, FDR). Attention and executive functions were mainly associated with edges related to parietal, temporal, prefrontal cortex, limbic lobe, insula and deep gray nuclei (Fig. 3). In the group of RRMS patients, correlations in frontoparietal connections, deep gray nuclei and insula remained significant (r between 0.40 and 0.54: *p* < 0.05, FDR) (see Supplementary material, Fig. S5).

More precisely, 160 connections correlated with zPASAT and 11 with zSDMT (*p* < 0.05, FDR). For PASAT z-score, the majority of correlations were found in the parietal, especially in bilateral precuneus and bilateral inferior parietal cortex, temporal, limbic lobe, deep gray nuclei, prefrontal, occipital cortex and in the insula (Fig. 4A). Most of the correlations were positive except in 17 connections. zSDMT positively correlated mainly with FA of prefrontal and deep gray nuclei edges and negatively correlated for connections between left pallidum and right pericalcarine cortices (Fig. 4B).

## 4 Discussion

Using improved tractography approaches, this study demonstrates structural connectivity changes in a cohort of MS participants at several levels, both in whole brain graph theory metrics and in GM nodal and WM edge integrity. Likewise, we identify strategic networks where connectivity is critical to preserve attention and executive performance in MS.

Previously, we demonstrated that anatomical information applied to postprocessing could improve reliability in the reconstruction of a specific tract (i.e. optic radiations) (Martínez-Heras et al., 2015). Here, we further support the use of this framework in structural connectivity analyses. We found that intra and inter-session ICC reproducibility index in high order probabilistic fiber tracking was high, showing a strong consistency between assessments (Landis and Koch, 1977). In addition, the use of AEC postprocessing was able to further improve the reproducibility and decrease the presence of aberrant streamlines, enhancing the anatomical reliability of the reconstruction (see Supplementary material, Figs. S2 and S3).

Healthy brain networks are characterized by short path lengths (associated with high global efficiency of information transfer), high clustering coefficient (associated with robustness to random error), and a modular community structure (Bullmore and Sporns, 2009). Graph theory studies have revealed the existence of hubs or groups of central nodes highly interconnected with other nodes of the same group and with lower level nodes, forming a rich and diverse community pattern (De Reus et al., 2014, Van den Heuvel and Sporns, 2011). In the present study, network hub distribution was mostly preserved in MS and hubs were found in medial parietal associative cortex, right insula and deep gray nuclei including thalamus. These regions are concordant with those described in healthy populations (Iturria-Medina et al., 2007, Van den Heuvel and Sporns, 2011) and in MS (Rocca et al., 2016, Shu et al., 2011). Nevertheless, we did not find hubs in the prefrontal areas, possibly due to differences in technical approaches of previous reports (Rocca et al., 2016, Shu et al., 2011). Remarkably, in our MS cohort, right pallidum and left insula became hubs of the network. Pallidum, a structure involved in movement control, showed increased FA connections with GM regions that have been related to somatosensory control (postcentral cortex) and cognition (isthmus of the cingulate and supramarginal cortices). Meanwhile, the insula is implicated in high level cognitive control and attentional processes. These nodes, especially pallidum, became more central and maintained their strength in a globally impaired network. This could reflect reorganization changes and high resilience of these areas to integrity damage. Despite differences in the methodology approach, the increased relevance of pallidum and insula have been described in other neurological and psychiatric diseases such as in early stages of Parkinson's disease (Sang et al., 2015) and schizophrenia (Bassett et al., 2008).

The present study shows decreased structural integration (global efficiency) and segregation impairment (decreased transitivity) in MS participants that worsen the efficiency in global and local transfer of information (Li et al., 2013, Shu et al., 2011). The decrease of WM FA is driven by the presence of focal lesions, but also related to microstructural impairment of normal appearing WM (Klistorner et al., 2015). Importantly, we also found increased FA of some edges connecting subcortical or cortical nodes, compared with HV. Indeed, increment of structural connectivity has been suggested in MS (Li et al., 2013) and related with WM lesion load (He et al., 2009). It can be present at early stages of disease (Fleischer et al., 2016), during recovery from relapses (Théaudin et al., 2012) and after a rehabilitation program (Ibrahim et al., 2011) evidencing dynamic variations of WM. Changes in the number of axons, axon diameter, the packing density of fibers, axon branching, axon trajectories, myelination, glial cell increase in number and size could underlie increased FA (Zatorre et al., 2012). However, histological studies are required to make direct links between imaging measures and underlying mechanisms.

The present analysis identifies strategic networks for maintaining attentional and executive functions and highlights the relevance of brain networks integrity in preserving cognition (Baggio et al., 2015, Crossley et al., 2013, Van den Heuvel and Sporns, 2011). In this cohort, we found that attention and executive functioning, mainly depending on PASAT performance, was associated with the structural integrity of the frontoparietal network, deep gray nuclei and insula, involving hub nodes. Both PASAT and SDMT are widely used tools to detect cognitive deterioration in MS (López-Góngora et al., 2015). However, PASAT seems more cognitively demanding and appears to be more sensitive to disease progression in a 5 years follow-up period (Borghi et al., 2016). Our results suggest that PASAT performance relies on the integrity of larger number of brain connections than SDMT (Fig. 3, Fig. 4), and support its suitability to detect cognitive deficits in mildly disabled patients. Loss of integrity in MS prompts brain synchronomy reduction and, thus cognitive performance worsening (Arrondo et al., 2009, Dineen et al., 2009, Rocca et al., 2016, Van Schependom et al., 2014), supporting the disconnection theory. Albeit WM burden seems the main driver of correlations between tissue integrity and cognition (Fjell et al., 2011), we cannot exclude that normal intersubject variability in FA influence our results (Dineen et al., 2009).

Functional MRI studies have found increased activity during PASAT performance in MS (Bonnet et al., 2010, Chiaravalloti et al., 2015, Ksiazek-Winiarek et al., 2015, Morgen et al., 2007, Rocca et al., 2016). Indeed, Morgen et al. (2007) found that low PASAT scores correlated with reduction of activation of anterior cingulate cortex, but also with enlarged activation of frontoparietal regions and more widely distributed cortical recruitment as an effort to maintain cognitive functioning. Similarly, our analysis revealed higher strength in right transverse temporal cortex, an area related to auditory codification stimuli (Audoin et al., 2005, Grasby et al., 1993), and FA increase in several connections (mainly connections from cingulate, frontal and occipital cortices) associated with cognitive performance. Taken together, our results and those from functional studies suggest both structural and functional brain reorganization in response to brain damage. However, the relation between structural and functional changes and disease progression is still controversial. Modifications of the cerebral architecture could be integral mechanisms to maintain optimal network functioning or reflect maladaptive changes promoting clinical dysfunction. Altogether, longitudinal studies are needed to understand the positive or deleterious consequences of brain reorganization (Bonnet et al., 2010).

The current study is not absent of limitations, and some are inherent in structural connectivity analyses in MS. Subjects with MS can present topology alterations of networks related to the spatial distribution of WM lesions that could affect tractography results. The use of high order probabilistic fiber tracking and AEC postprocessing partially resolves this handicap by improving tract reconstruction in the presence of lesions (Bucci et al., 2013, Mormina et al., 2015) and decreasing low confidence and spurious streamlines (Martínez-Heras et al., 2015). Further research on tractography methodology to solve the effect of lesions in tract reconstruction is warranted. Moreover, our cohort is composed by MS subjects with mild cognitive disability (only 10% of them had zAttention 1.5 SD below normative data), which could limit the results related to cognitive impairment but enables the study of structural plasticity changes. Finally, we are aware that our set of MS participants was mainly composed by relapsing remitting subjects; hence our results will mainly apply to this form of the disease.

## 5 Conclusions

In conclusion, we found that, in MS, structural brain network is less efficient due to widespread impairment of WM connections and GM structures. The observed increased connectivity of some edges suggests the presence of reorganization mechanisms at the structural level. However, further longitudinal studies combining structural and functional approaches are needed to better understand the consequences of dynamic network changes. Finally, attention and executive performance relates to frontoparietal networks, deep gray nuclei and insula. These results support the relevance of network integrity to maintain optimal cognitive skills.

The following are the supplementary data related to this article.

## Figures and Tables

**Table 1:** Clinical and demographic characteristics of the subjects included in the study.

	MS patients (*n* = 72)	Healthy volunteers (*n* = 38)	*p* value
Female (N, %)	47 (65.3)	21 (55.3)	0.30^a^
Age (years)	38.95 (9.55)	40.01 (11.14)	0.60^b^
Type of MS:			
RRMS	66 (94.5)	n/a	n/a
SPMS	6 (5.5)		
Lesion volume (cm^3^)	5.73 (8.42)	n/a	n/a
Gray matter volume (cm^3^)	625.1 (66.7)	641.7 (68.1)	0.22^b^
Disease duration, years	6.54 (7.34)	n/a	n/a
Median EDSS score (range)	2.0 (0–6.0)	n/a	n/a
zPASAT score	− 0.43 (0.17)	n/a	n/a
zSDMT score	0.14 (1.19)	n/a	n/a
zAttention score	− 0.14 (1.10)	n/a	n/a

Continuous variables are given as mean (standard deviation). EDSS = Expanded Disability Status Scale; n/a = not applicable; PASAT = Paced Auditory Serial Addition Test; RRMS = relapsing remitting multiple sclerosis; SDMT = Symbol Digit Modalities Test; SPMS = secondary progressive multiple sclerosis.

a Chi square test.

b Student's *t*-test for independent samples.

**Table 2:** Whole network graph theory properties.

	MS patients (*n* = 72)	Healthy volunteers (*n* = 38)	*p* value
Strength	22.64 (2.20)	23.63 (2.24)	0.028
Transitivity	0.33 (0.02)	0.34 (0.02)	0.001⁎
Global efficiency	0.36 (0.02)	0.38 (0.02)	< 0.001⁎
Assortativity	− 0.02 (0.02)	− 0.03 (0.02)	0.193
Path length	3.05 (0.17)	2.92 (0.15)	< 0.001⁎
Betweenness centrality	32.58 (4.55)	32.47 (5.32)	0.906

Statistics by Student's *t*-test for independent samples. Variables are given as mean (standard deviation).

⁎ Statistical significance at *p* < 0.008 using Bonferroni correction.
